# HRS Facilitates Newcastle Disease Virus Replication in Tumor Cells by Promoting Viral Budding

**DOI:** 10.3390/ijms251810060

**Published:** 2024-09-19

**Authors:** Yu Chen, Chunxuan Wang, Shunlin Hu, Xiufan Liu

**Affiliations:** 1 Animal Infectious Disease Laboratory, College of Veterinary Medicine, Yangzhou University, Yangzhou 225012, China; 2 Jiangsu Co-Innovation Center for Prevention and Control of Important Animal Infectious Diseases and Zoonosis, Yangzhou University, Yangzhou 225012, China; 3 Jiangsu Key Laboratory of Zoonosis, Yangzhou University, Yangzhou 225012, China

**Keywords:** NDV, ESCRT, HRS, viral replication, viral budding

## Abstract

Newcastle disease virus (NDV) is a highly pathogenic avian infectious disease agent and also a promising oncolytic virus with broad application prospects. The Endosomal Sorting Complex Required for Transport (ESCRT) machinery has been increasingly recognized for its crucial role in the life cycles of enveloped viruses, influencing processes such as viral entry, replication, and budding. In this study, we employed an RNA interference screening approach to identify key ESCRT components that regulate NDV replication in tumor cells. qPCR, immunofluorescence, and Western blot assays demonstrated that knockdown of HRS, CHMP4A, CHMP4B, and CHMP4C significantly impaired NDV replication in HeLa cells, with HRS exhibiting the most pronounced inhibitory effect. Additionally, HRS knockout significantly inhibited viral budding and suppressed NDV-induced cell death in HeLa cells. Notably, NDV infection was shown to significantly upregulate HRS gene and protein expression in a time-dependent manner. In conclusion, this study systematically identifies critical ESCRT components involved in NDV replication within tumor cells, with a particular focus on the role of HRS in promoting NDV’s replication by promoting viral budding, offering new insights for the development of NDV-based oncolytic therapies.

## 1. Introduction

Newcastle disease (ND) is an acute, highly contagious infectious disease affecting chickens and various avian species. Due to its significant economic impact on the global poultry industry, the World Organization for Animal Health designates it as a notifiable animal disease [1]. As the causative agent of ND, Newcastle disease virus (NDV) infects not only the majority of avian species but also demonstrates a unique capability to selectively target tumor cells and induce cell death through various mechanisms [2,3,4]. This characteristic positions it as a natural oncolytic virus with significant potential in clinical oncology [5]. NDV belongs to the class of single-stranded negative-sense RNA viruses. Its viral genome is approximately 15.1 kb in length, encoding NP (Nucleocapsid protein), P (Phosphate protein), M (Matrix Protein), F (Fusion protein), HN (Haemagglutinin Neuraminidase protein), and L (Large protein) proteins sequentially from the 3′ to 5′ end [6]. Additionally, during transcription, the P gene undergoes “RNA editing”, resulting in the production of two non-structural proteins: V and W proteins [7].

Similar to most enveloped viruses, NDV infection begins with the binding of the viral HN protein to specific receptors on the host–cell membrane [4]. This binding triggers a conformational change in the F protein, facilitating fusion of the viral envelope with the host–cell plasma membrane, ultimately releasing the NDV nucleocapsid into the cytoplasm [8]. Within the cytoplasm, the NDV nucleocapsid undergoes transcription and translation to produce viral proteins while concurrently replicating the viral genome to generate progeny viral genomes. Subsequently, viral proteins and genomes assemble on the inner side of the host–cell membrane and are released through budding. Despite the identification of certain factors, there remains a significant gap in research on host cellular factors involved in NDV infection, particularly during viral budding.

The Endosomal Sorting Complex Required for Transport (ESCRT) in mammalian cells comprises five main complexes: ESCRT-0, I, II, III, and vacuolar protein sorting-associated protein 4 (VPS4) [9,10,11]. Additionally, it includes several accessory proteins, such as ALG-2-Interacting Protein X (Alix) [12,13]. ESCRT-0, I, and II complexes are responsible for recruiting and transporting membrane proteins and viral particles [14,15,16]. ESCRT-III possesses scission activity, facilitating the initiation of vesicle constriction and membrane scission from the target membrane [17,18]. VPS4, an AAA ATPase, hydrolyzes ATP, catalyzing the disassembly of ESCRT complexes for recycling and reuse [19,20].

Within eukaryotic cells, ESCRT functions include lipid bilayer membrane scission [21] and involvement in various metabolic processes such as cytoplasmic segregation [22], cellular autophagy [23,24], membrane fission and remodeling [25], and plasma membrane repair [26]. Moreover, the roles of ESCRT complexes in various viral entry, replication, and budding processes have gradually been elucidated [27,28]. For instance, the ESCRT-I complex component TSG101 contributes to porcine reproductive and respiratory syndrome virus (PRRSV) virion formation through interaction with the viral N protein along with the early secretory pathway [29,30]. TSG101 also plays novel dual roles in the entry and replication of classical swine fever virus [31]. Additionally, ALIX and charged multivesicular body protein 4A (CHMP4A) are required during flavivirus infection, though detailed mechanisms remain to be fully elucidated [32]. Furthermore, due to ESCRT’s role in the budding process of the SARS-CoV-2 virus, researchers have developed a self-assembling and budding SARS-CoV-2 mRNA vaccine by inserting an ESCRT and ALIX-binding region into the SARS-CoV-2 spike cytoplasmic tail [33].

Although the hijacking of ESCRT-related subunits by various viruses to promote viral replication has been widely documented, the role of ESCRT in NDV infection of tumor cells remains poorly understood. In this study, we conducted a systematic small interfering RNA (siRNA) screening assay and identified specific ESCRT components crucial for NDV infection in HeLa cells. Among these components, hepatocyte growth factor-regulated tyrosine kinase substrate (HRS) was selected for further investigation regarding its involvement in NDV infection through multiple approaches. We observed an upregulation of HRS expression upon NDV infection, and further experiments revealed that HRS promotes NDV budding, thereby facilitating NDV replication in tumor cells. This discovery further enriches our understanding of the oncolytic mechanisms of NDV and provides a new target for NDV-based oncolytic therapy.

## 2. Results

### 2.1. Identification of Key ESCRT Subunits Regulating NDV Replication in HeLa Cells

**Figure 1:**
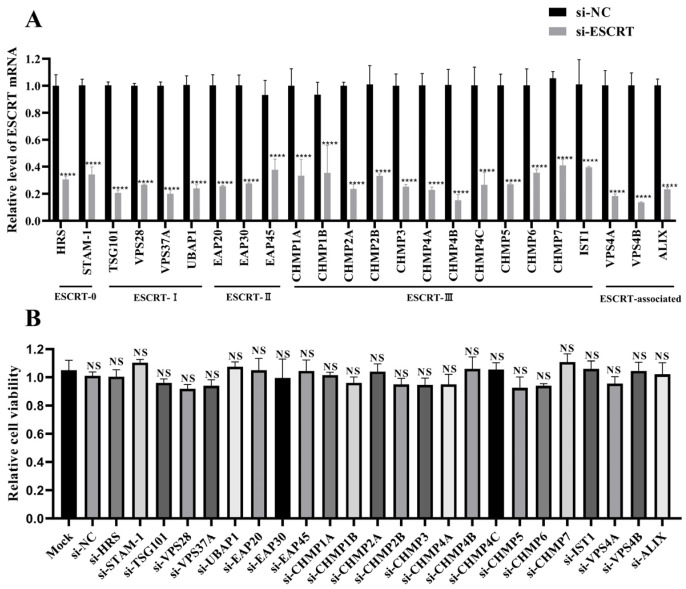
Assessment of siRNA knockdown efficiency and cytotoxicity. (**A**) Detection of siRNA knockdown efficiency. HeLa cells were transfected with siRNAs targeting ESCRT subunits or si-NC for 48 h. The mRNA levels of the specified ESCRT subunits were quantified by qPCR and normalized to GAPDH using the 2^−ΔΔCt^ method. (**B**) siRNA cytotoxicity assessment. HeLa cells cultured in 96-well plates were transfected with the indicated siRNAs, and cell viability was assessed using a CCK-8 assay at 48 h post-transfection (hpt). Data are presented as mean ± standard deviations (SDs) from three independent experiments. Statistical analysis was performed using a two-way ANOVA for the qPCR assay and a one-way ANOVA for the CCK-8 assay. NS means no significant difference, **** *p* < 0.0001.

To identify ESCRT subunits that regulate NDV replication in tumor cells, 24 siRNAs targeting different ESCRT subunits were designed and synthesized. The knockdown efficiency of these siRNAs was then assessed using qPCR. It was shown that all siRNAs achieved a knockdown efficiency of approximately 60–80% of mRNA levels in HeLa cells (Figure 1A). Cell viability assays indicated that none of the siRNAs were toxic to HeLa cells, as cell viability measured by the CCK-8 assay was not significantly different from the siRNA negative control (si-NC) group (Figure 1B).

**Figure 2:**
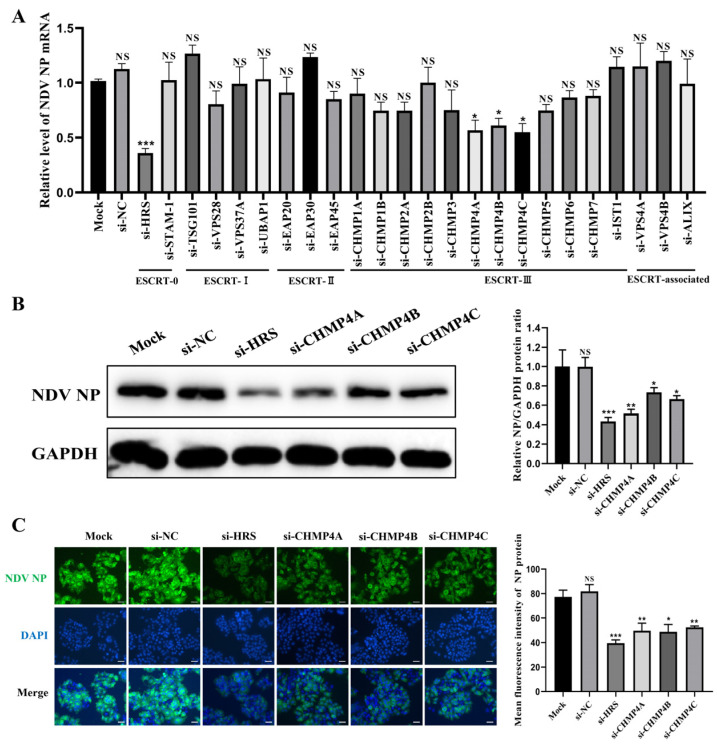
Identification of key ESCRT subunits regulating NDV replication. (**A**–**C**) HeLa cells were transfected with the indicated siRNAs or mock transfected for 48 h and then infected with the Herts/33 strain at a multiplicity of infection (MOI) of 1 for 12 h. (**A**) The mRNA levels of the NP gene were quantified by qPCR and normalized to GAPDH using the 2^−ΔΔCt^ method. (**B**) The NP protein levels were detected by Western blot. The relative intensity of the NP protein was quantified using GAPDH as a normalization control, as shown in the right panel. (**C**) The NP protein was detected by immunofluorescence assay (IFA). The total fluorescence intensity of the NP protein was calculated using ImageJ software (version number: 1.53s) and displayed in the right panel. Scale bars represent 50 µm. Error bars represent SDs from triplicate analyses of three independent experiments. Representative images for Western blot and IFA are shown. Statistical significance was assessed using one-way ANOVA. NS means no significant difference, * *p* < 0.05, ** *p* < 0.01, *** *p* < 0.001.

Next, HeLa cells were transfected with these siRNAs or si-NC, respectively. At 48 h post-transfection (hpt), cells were infected with NDV at a multiplicity of infection (MOI) of 1. At 12 h post-infection (hpi), the levels of the viral NP mRNA were measured using qPCR. As shown in Figure 2A, the knockdown of ESCRT-I, ESCRT-II, and ESCRT-associated subunits had minimal impact on the viral NP mRNA. In contrast, the knockdown of ESCRT-0 component HRS and ESCRT-III components CHMP4A, CHMP4B, and CHMP4C significantly decreased the mRNA level of the NP gene.

To further validate these findings, HeLa cells were transfected with siRNAs targeting HRS, CHMP4A, CHMP4B, and CHMP4C, and their effects on NDV replication were assessed by Western blot and immunofluorescence assay (IFA). As shown in Figure 2B,C, the knockdown of any of these genes inhibited NDV replication to varying degrees, with HRS knockdown exhibiting the most significant inhibitory effect. Taken together, these results illustrate that certain components of the ESCRT system are important for NDV replication in tumor cells.

### 2.2. HRS Positively Regulates NDV Replication in HeLa Cells

HRS is a critical component of ESCRT-0, known to play a significant regulatory role in facilitating the degradation of membrane proteins and regulating the cytoplasmic lysosomal pathway [34]. Additionally, in recent years, an increasing body of research indicates that HRS is hijacked by many viruses to promote their replication [35,36,37]. Therefore, this study was focused on exploring the role of HRS in NDV replication in tumor cells.

**Figure 3:**
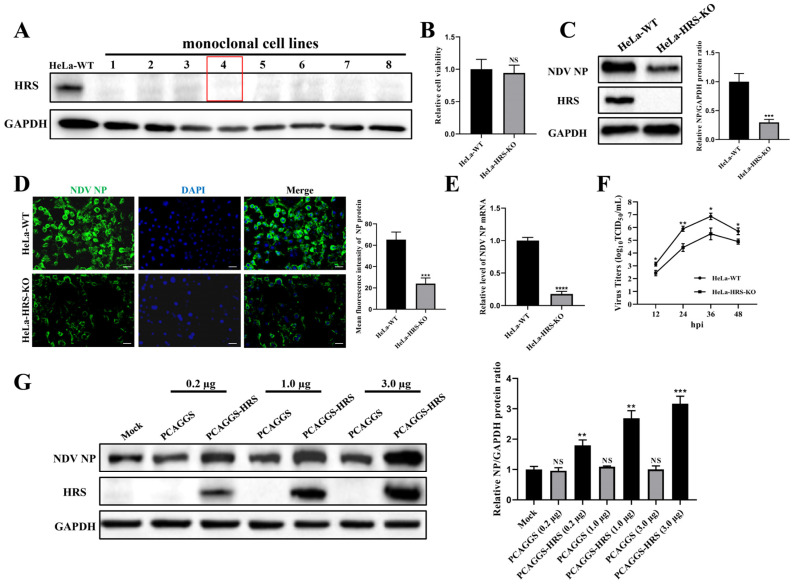
HRS positively regulates NDV replication in HeLa cells. (**A**) Validation of HRS knockout cell lines. HeLa cell lines with HRS gene knockout were created using CRISPR/Cas9 technology, as detailed in the Materials and Methods section. The knockout of HRS was confirmed by Western blot. HeLa-WT were used as a control. The red box highlights the cell line used in this study, named HeLa-HRS-KO. (**B**) Cytotoxicity evaluation of HRS knockout. The cell viability of HeLa-WT and HeLa-HRS-KO cells was assessed using a CCK-8 assay. (**C**–**F**) Impact of HRS knockout on NDV replication. HeLa-WT and HeLa-HRS-KO cells were infected with the Herts/33 strain at an MOI of 1. (**C**) The viral NP protein was detected by Western blot, and its relative intensity was quantified using ImageJ software and normalized to GAPDH, as shown in the right panel. (**D**) The viral NP protein was detected by IFA. The numbers of NP-expressing (green) and DAPI-stained (blue) cells were counted using ImageJ software. The infection rate was calculated as the ratio of NP-expressing cells to DAPI-stained cells, shown in the right panel. Scale bars represent 50 µm. (**E**) The mRNA levels of the NO gene were quantified by qPCR and normalized to GAPDH using the 2^−ΔΔCt^ method. (**F**) Extracellular progeny virus titers in the cell supernatant were measured using a TCID_50_ assay. (**G**) Effect of HRS overexpression on NDV replication. HeLa-HRS-KO cells were transfected with the PCAGGS-HRS plasmid or vector control at specified doses for 48 h. The cells were then infected with the Herts/33 strain at an MOI of 1. The viral NP protein was detected by Western blot, and its relative intensity was quantified using ImageJ software and normalized to GAPDH, as shown in the right panel. Error bars represent SDs from triplicate analyses of three independent experiments. Representative images for Western blot and IFA are shown. Statistical significance was assessed using Student’s *t* tests for (**B**–**E**), one-way ANOVA for (**G**) and two-way ANOVA for (**F**). NS means no significant difference, * *p* < 0.05, ** *p* < 0.01, *** *p* < 0.001.

The HRS knockout HeLa cell line was generated using CRISPR-Cas9 technology. After puromycin selection and subcloning steps, a total of eight monoclonal cell lines were obtained. The complete absence of HRS protein expression was confirmed by Western blot analysis (Figure 3A). Among these, the fourth monoclonal cell line was used for this study and was designated as HeLa-HRS-KO. The impact of HRS knockout on HeLa cell viability was accessed by CCK-8 assay. The result indicates that HRS knockout alone has no significant effect on cell viability (Figure 3B). Using this model, the effects of HRS knockout on NDV replication were examined.

According to the Western blot and IFA results shown in Figure 3C,D, the expression level of the viral NP protein at 12 hpi was significantly reduced by HRS knockout. Moreover, the levels of viral NP mRNA at 12 hpi were markedly decreased by HRS knockout, as determined by qPCR (Figure 3E). Aligning with these observations, virus growth curves, measured by TCID_50_ assay, also revealed that HRS knockout significantly reduced NDV replication in HeLa cells (Figure 3F).

To further verify HRS’s role in regulating NDV replication, an HRS overexpression plasmid named PCAGGS-HRS was constructed. It was demonstrated that reintroducing HRS protein into HeLa-HRS-KO cells significantly elevated the expression of the viral NP protein in a dose-dependent manner when compared to cells where HRS was knocked out (Figure 3G). These findings collectively demonstrate that HRS positively regulates NDV replication in HeLa cells.

### 2.3. Knockout of HRS Significantly Suppresses NDV-Induced Cell Death of HeLa Cells

**Figure 4:**
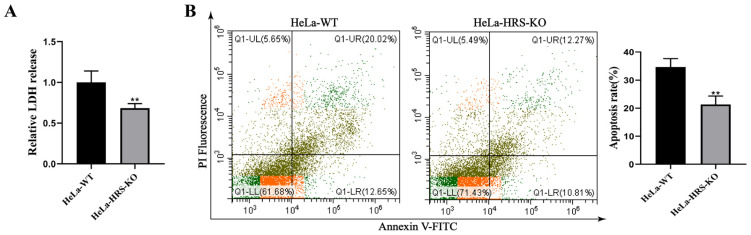
Knockout of HRS significantly suppresses NDV-induced cell death of HeLa cells. (**A**) Detection of LDH release in NDV-infected HeLa-WT or HeLa-HRS-KO cells. HeLa-WT and HeLa-HRS-KO cells were infected with the Herts/33 strain at an MOI of 1. The abundance of LDH was detected at 12 h post-infection (hpi). (**B**) Detection of apoptosis in NDV-infected HeLa-WT or HeLa-HRS-KO cells. HeLa-WT and HeLa-HRS-KO cells were infected with the Herts/33 strain at an MOI of 1. The apoptosis level was measured by AnnexinV-FITC/PI staining using flow cytometry at 12 hpi, and quantitation of the apoptosis rate was shown in the right panel. Error bars represent SDs from triplicate analyses of three independent experiments. Representative images for apoptosis detection are shown. Statistical significance was assessed using Student’s *t* tests for (**A**,**B**). ** *p* < 0.01.

Inducing tumor cell death is a crucial mechanism through which NDV exerts its oncolytic effects [38]. To further explore the role of HRS in NDV’s oncolytic activity, we conducted experiments to assess the impact of HRS knockout on NDV-induced cell death. Lactate dehydrogenase (LDH), typically contained within cells, is released into the extracellular environment as a marker of membrane-disruptive cell death. As shown in Figure 4A, NDV-induced LDH release was significantly lower in HeLa-HRS-KO cells compared to HeLa-WT cells, indicating that HRS knockout inhibits NDV-induced tumor cell death. Furthermore, flow cytometry analysis revealed that HRS knockout significantly reduced NDV-induced apoptosis (Figure 4B). Given that cell death is a primary pathway through which NDV mediates its oncolytic effects, these findings suggest that HRS knockout impairs NDV’s oncolytic activity.

### 2.4. NDV Infection Significantly Upregulates the Expression of HRS

**Figure 5:**
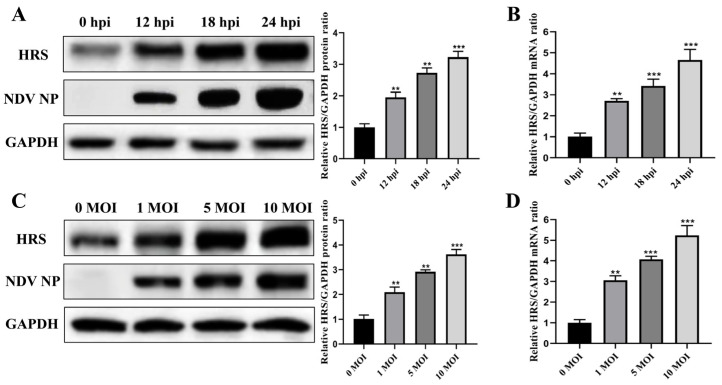
NDV infection significantly upregulates the expression of HRS. (**A**,**B**) HeLa cells were infected with Herts/33 at an MOI of 1. The protein and mRNA levels of endogenous HRS were then detected by Western blot (**A**) and qPCR (**B**) at indicated time points (0, 12, 18, and 24 hpi). (**C**,**D**) HeLa cells were infected with Herts/33 at indicated MOIs (0.01, 0.1, and 1). The protein and mRNA levels of endogenous HRS were then detected by Western blot (**C**) and qPCR (**D**) at 12 hpi. The grayscale of HRS was quantified using ImageJ software and normalized to GAPDH, as shown in the right panel of corresponding image. The mRNA levels of the HRS gene were also normalized to GAPDH using the 2^−ΔΔCt^ method. Error bars represent SDs from triplicate analyses of three independent experiments. Representative images for Western blot are shown. Statistical significance was assessed using one-way ANOVA. ** *p* < 0.01, *** *p* < 0.001.

To further explore the involvement of HRS in NDV replication, we investigated the expression level of HRS during NDV infection. HeLa cells were infected with Herts/33 at an MOI of 1 for 12, 18, and 24 h, or infected with Herts/33 at MOIs of 0.01, 0.1, and 1 for 12 h. The protein and mRNA levels of HRS were then detected by Western blot and qPCR assays. As shown in Figure 5A,B, the protein and mRNA levels of HRS increased in a time-dependent manner following NDV infection. Specifically, the HRS protein level was upregulated approximately 3-fold, and the mRNA level was upregulated approximately 5-fold at 24 hpi. Furthermore, after 12 h of infection with different doses of NDV, the protein and mRNA levels of HRS increased in a dose-dependent manner (Figure 5C,D). These results indicate that NDV infection promotes the expression of HRS, which may further enhance NDV replication in HeLa cells.

### 2.5. HRS Does Not Affect NDV Attachment and Internalization in HeLa Cells

Like most enveloped viruses, the life cycle of NDV can be briefly divided into the following stages: attachment, internalization, RNA replication, viral protein translation, and progeny virions assembly and budding. Having established that HRS promotes NDV replication in HeLa cells, we further investigated how HRS regulates NDV replication by focusing on which specific stage of the viral life cycle it affects.

**Figure 6:**
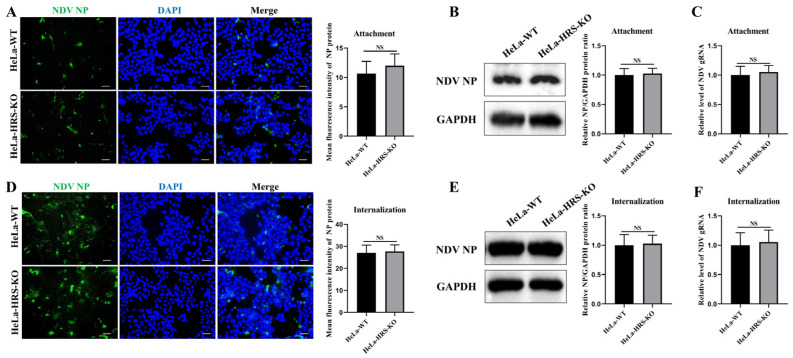
HRS does not affect NDV attachment and internalization in HeLa cells. (**A**–**C**) Knockout of HRS does not affect NDV attachment. HeLa-WT and HeLa-HRS-KO cells were infected with Herts/33 at an MOI of 10 and cultured at 4 °C for 1 h. After adsorption, unbound viruses were extensively washed away with ice-cold phosphate-buffered saline (PBS). (**A**) Cell-attached NDV virions were assessed with a mouse anti-NP monoclonal antibody (green) by IFA. (**B**) NP protein of cell-attached NDV virions was quantified by Western blot. (**C**) Genomic RNA of cell-attached NDV virions was quantified by qPCR. (**D**–**F**) Knockout of HRS does not affect NDV internalization. HeLa-WT and HeLa-HRS-KO cells were infected with Herts/33 at an MOI of 10 and incubated at 4 °C for 1 h. Unbound NDV virions were washed away with ice-cold PBS, and the cells were switched to 37 °C for 1 h. Internalized virions were quantified by IFA (**D**), Western blot (**E**), and qPCR (**F**) as described above. Total fluorescence intensity of NP protein in (**A**,**D**) was calculated using ImageJ software and shown in the right panel. Relative intensity of NP protein in (**B**,**E**) was normalized to GAPDH and shown in the right panel. Viral genomic RNA was also normalized to GAPDH using the 2^−ΔΔCt^ method. Error bars represent SDs from triplicate analyses of three independent experiments. Representative images for IFA and Western blot are shown. Scale bars for IFA images represent 50 µm. Statistical significance was assessed using Student’s *t* tests. NS means no significant difference.

HeLa-HRS-KO and wild-type HeLa (HeLa-WT) cells were inoculated with NDV at an MOI of 10 and incubated at 4 °C for 1 h to facilitate viral attachment. Following the adsorption phase, unbound viruses were thoroughly washed away, and the bound viruses were visualized using IFA to analyze viral attachment. As shown in Figure 6A, there was no significant difference in the amount of bound NDV virions between HeLa and HeLa-HRS-KO cells, indicating that HRS does not influence the initial attachment of NDV to host cells. Additionally, cell-bound viruses were assessed by Western blot and qPCR assays to detect NP protein and viral genomic RNA levels, respectively. As illustrated in Figure 6B,C, knockout of HRS had no significant impact on the abundance of viral NP protein or genomic RNA levels, corroborating the fluorescence intensity data obtained from the IFA. These consistent findings confirm that HRS does not affect the viral attachment stage during NDV’s life cycle.

Subsequently, we investigated whether HRS plays a role in NDV internalization. HeLa-WT and HeLa-HRS-KO cells were inoculated with NDV at an MOI of 10 and incubated at 4 °C for 1 h to allow attachment, followed by incubation at 37 °C for 1 h to facilitate internalization. The intracellular virions were then measured using IFA, Western blot, and qPCR assays. As shown in Figure 6D–F, HRS knockout had no significant effect on the abundance of intracellular virions, NP protein, or viral genomic RNA levels. These results collectively suggest that HRS does not impact the viral internalization stage during NDV’s life cycle.

### 2.6. HRS Promotes NDV Replication in HeLa Cells by Enhancing Viral Budding

**Figure 7:**
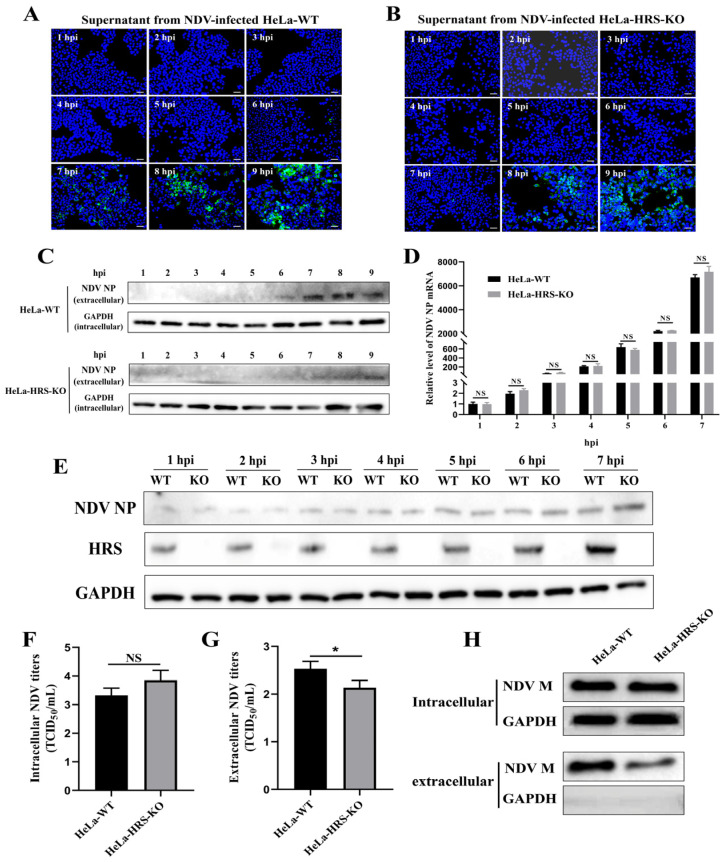
HRS promotes NDV replication in HeLa cells by enhancing viral budding. (**A**,**B**) Determination of the first life cycle of NDV. HeLa-WT and HeLa-HRS-KO cells were infected with Herts/33 at an MOI of 1, and the cellular supernatant was harvested every 1 h until 9 hpi. Fresh DF-1 cells grown in 24-well plates were infected with the collected supernatants for 12 h. IFA was performed to detect the fluorescence signal of the virus stained with a mouse anti-NP monoclonal antibody (green). Nuclei were stained with DAPI (blue). Scale bars represent 50 µm. (**C**) The proteins in the supernatants were concentrated using TCA precipitation. Proteins from each sample were used for Western blot to detect the abundance of progeny virus with a mouse anti-NP monoclonal antibody. The protein level of intracellular GAPDH was used as a control. (**D**,**E**) HRS does not impact viral RNA replication or protein translation. HeLa-WT and HeLa-HRS-KO cells were infected with Herts/33 at an MOI of 1, and cells were harvested every 1 h until 7 hpi. (**D**) The NP mRNA levels in the cells were detected by qPCR and normalized to GAPDH using the 2^−ΔΔCt^ method. (**E**) The NP protein levels in the cells were detected by Western blot. (**F**,**G**) HRS promotes NDV replication by enhancing viral budding. The HeLa-WT and HeLa-HRS-KO cells were infected with Herts/33 at an MOI of 1 for 7 h. The infected cells and supernatants were harvested separately for detection of the intracellular (**F**) and extracellular (**G**) progeny virus titers by assessing TCID_50_. (**H**) Evaluation of the impact of HRS knockout on NDV M protein-mediated budding. HeLa-WT and HeLa-HRS-KO cells were transfected with plasmids expressing Flag-tagged M protein for 48 h. The cells and culture supernatants were collected, and the supernatants were purified and concentrated as described in the Materials and Methods section. M protein expression in the cells and culture supernatants was then detected by Western blot. Error bars represent SDs from triplicate analyses of three independent experiments. Representative images for IFA and Western blot are shown. Statistical significance was assessed using two-way ANOVA for (**D**) and Student’s *t* tests for (**F**,**G**). NS means no significant difference, * *p* < 0.05.

Having determined that HRS does not influence viral attachment or internalization stages, we next focused on understanding if HRS regulates NDV replication by affecting viral RNA replication, viral protein translation, or progeny virions assembly and budding. To this end, we determined the duration of the NDV first life cycle by detecting the earliest time points at which progeny virions appeared in the supernatant of an NDV-infected cell. In brief, supernatants in NDV-infected HeLa and HeLa-HRS-KO cells were collected at indicated time points and were used to infect DF-1 cells, a continuous line of fibroblast cells derived from chicken embryos. DF-1 cells are commonly used in avian virology research due to their susceptibility to various avian viruses, including NDV [39,40]. Consequently, if NDV virions were present in the supernatant, they would be detectable in DF-1 cells after 12 h of proliferation. As shown in Figure 7A, progeny virions were detectable in the supernatant of NDV-infected HeLa cells at approximately 7 hpi, whereas in NDV-infected HeLa-HRS-KO cells, progeny virions were detectable at around 8 hpi (Figure 7B). These results were further confirmed by Western blot analysis (Figure 7C), indicating that the knockout of HRS prolongs the first life cycle of NDV in HeLa cells.

Considering that the first life cycle of NDV in HeLa cells is approximately 7 h, we subsequently evaluated the role of HRS in NDV genomic RNA replication and NP protein translation within 7 hpi. Knockout of HRS did not alter the abundance of intracellular viral genomic RNA (Figure 7D) or the intracellular viral NP protein (Figure 7E) within 7 hpi, demonstrating that HRS does not impact viral RNA replication or protein translation during NDV’s first life cycle.

Building on these results, we further investigated whether the knockout of HRS extends NDV’s first life cycle by affecting progeny virion assembly and/or budding. To distinguish between the roles of HRS in NDV assembly and budding, we measured both intracellular and extracellular viral titers at 7 hpi, as outlined in a previous study [29]. As shown in Figure 7F,G, HRS knockout slightly increased intracellular viral titers at 7 hpi, suggesting that HRS knockout does not significantly impact NDV assembly within the cell. However, HRS knockout significantly reduced extracellular viral titers at 7 hpi, indicating a notable inhibition of NDV budding.

Previous studies have demonstrated that the NDV M protein is crucial for NDV budding, with its expression alone sufficient to form virus-like particles (VLPs) that are released into the supernatant [41]. To assess the impact of HRS knockout on NDV M protein-mediated budding, we transfected plasmids expressing Flag-tagged M protein into both HeLa-WT and HeLa-HRS-KO cells. At 48 hpt, cells and culture supernatants were collected, and the supernatants were purified and concentrated. M protein expression in the cells and culture supernatants was then detected by Western blot. As shown in Figure 7H, intracellular M protein expression levels were comparable between HeLa-WT and HeLa-HRS-KO cells. The GAPDH signal was nearly undetectable in the concentrated supernatant, confirming the absence of dead cells or debris. Thus, the M protein detected in the supernatant reflects its incorporation into VLPs. The results indicate that HRS knockout significantly reduces the amount of M protein in the supernatant, suggesting that HRS is critical for efficient NDV M protein-mediated budding.

In conclusion, these data suggest that HRS promotes NDV replication in HeLa cells by enhancing viral budding.

## 3. Discussion

The ESCRT pathway was initially identified through genetic analyses in yeast, which defined the factors required to target membrane proteins for degradation within vacuoles. This discovery has since evolved, with the interplay between the ESCRT and virus replication garnering significant interest due to the crucial role that ESCRT components play in various stages of the viral life cycle [27,42]. In addition to infecting most poultry, NDV can also specifically infect tumor cells, making it a promising oncolytic virus [5]. In this study, we aimed to identify and explore the role of ESCRT components in the oncolytic process of NDV. To this end, HeLa cells were chosen, as they are widely used in assessing the oncolytic activity of oncolytic viruses in vitro, including studies involving NDV [43,44,45], adenovirus [46,47] and herpes simplex virus [48,49]. Our findings provide new insights into the mechanisms through which NDV manipulates host cellular machinery to enhance its replication, specifically highlighting the role of ESCRT in viral budding during NDV’s oncolytic process.

Through comprehensive siRNA screening, we identified that the knockdown of certain ESCRT components, notably HRS (ESCRT-0) and CHAMP4A, 4B, and 4C proteins (ESCRT-III), significantly inhibited NDV replication in HeLa cells. This aligns with previous studies demonstrating the essential roles of ESCRT components in the life cycles of various enveloped viruses [27]. For instance, TSG101, a component of ESCRT-I, is known to be critical for the replication of porcine reproductive and respiratory syndrome virus by facilitating virion formation through interaction with the viral N protein [29]. Similarly, the ESCRT components ALIX and CHMP4A have been identified as crucial for the virus replication of flaviviruses [32]. Despite these contributions, significant gaps in our understanding of ESCRT pathway recruitment and function remain, particularly regarding the specificity of different ESCRT components to various viruses.

Viruses must navigate the formidable barriers posed by cell membranes to spread infection, necessitating complex strategies for both entry and exit. While most enveloped viruses encode their own membrane fusion proteins to mediate entry, many viruses harness the cellular ESCRT machinery to effect membrane fission during budding. In the context of NDV, existing literature has indicated that the ESCRT-III complexes, particularly CHMP4B, are involved in viral budding [50,51]. However, these studies have primarily been conducted in avian-derived cells, or Vero cells. Our study expands on these findings by identifying ESCRT components that regulate NDV replication in tumor cells. We demonstrate that HRS facilitates NDV replication by enhancing viral budding rather than affecting earlier stages of the viral life cycle.

HRS, ubiquitously expressed in various cell types, is a key component of the ESCRT-0 complex. It recognizes and binds ubiquitinated membrane proteins, recruits them to early endosomes, and lays the groundwork for subsequent protein degradation processes [52]. In addition, since the initial discovery that human immunodeficiency virus type 1 (HIV-1) usurps the HRS to bud from the plasma membrane [37], the role of HRS in viral budding has also been observed in other viruses. For instance, HRS has been shown to play a dual role in hepatitis B virus transcription and naked capsid secretion by interaction with both viral nucleocapsids and surface antigens [53]. In addition, during hepatitis C virus replication, HRS play a significant role in the viral budding through the exosomal secretion pathway [36]. In our present study, we also found that HRS facilitates NDV replication in HeLa cells by promoting viral budding, indicating that HRS may function as a common factor during various viral budding.

Interestingly, despite the inhibition of viral budding in HeLa-HRS-KO cells, we observed no significant increase in intracellular levels of the NDV M protein, as might be expected when the budding process is blocked (Figure 7H). Similar results have also been reported in other studies on NDV M protein-mediated budding [54,55].We propose two potential factors that may account for this discrepancy. First, upon inhibition of viral budding, the majority of intracellular M protein may remain bound to the inner side of the plasma membrane. Due to the hydrophobic nature of the membrane, it is possible that membrane-bound M protein was not efficiently extracted during sample preparation for Western blot assay. Second, the absence of HRS may directly or indirectly impact the stability of NDV M protein, potentially leading to its degradation within HeLa-HRS-KO cells. Further investigation into these mechanisms is required to fully understand the intracellular behavior of M protein under HRS knockout conditions.

Our study also reveals that NDV infection significantly upregulates HRS expression in a time- and dose-dependent manner. This upregulation likely enhances NDV replication by increasing the availability of HRS for the budding process. Virus-induced regulation of host factors is a common strategy employed by viruses to optimize their replication. The upregulation of HRS by NDV adds to this body of knowledge, indicating that NDV can manipulate host ESCRT machinery to facilitate its replication. The process of virus budding is typically integrated with virion assembly, prompting most viruses to employ their structural proteins to attract the ESCRT pathway [56]. However, the mechanisms by which NDV infection upregulates HRS expression and promotes NDV budding require further study. Future investigations should also consider whether similar upregulation mechanisms apply to other oncolytic viruses, potentially broadening the therapeutic applications of this strategy.

NDV’s oncolytic properties, particularly its ability to selectively infect and kill tumor cells, position it as a promising candidate for cancer therapy. In our study, we also found that HRS enhances NDV-induced tumor cell death, offering valuable insights for the development of oncolytic therapies. Future research should focus on elucidating the precise molecular mechanisms by which HRS promotes viral budding. Targeting HRS or other ESCRT components could modulate NDV replication, providing a novel approach to enhance the efficacy of NDV-based oncolytic therapies. Additionally, understanding the molecular interactions between NDV and ESCRT components may aid in designing engineered NDV strains with optimized oncolytic activity.

In summary, this study highlights the pivotal role of ESCRT components, especially HRS, in the replication and oncolytic effect of NDV in tumor cells. By facilitating the budding of progeny virions, HRS enhances NDV replication and promotes cell death in tumor cells. Although the precise molecular and cellular mechanisms by which NDV infection upregulates HRS expression to promote viral budding remain to be elucidated, our data suggest that exploring ESCRT components as therapeutic targets in oncolytic virotherapy holds promise for enhancing the efficacy of NDV-based cancer treatments. Furthermore, this research opens avenues for investigating the role of ESCRT components in other oncolytic viruses, potentially leading to broader applications in virotherapy.

## 4. Materials and Methods

### 4.1. Cell Lines and Virus

HeLa (human cervical cancer cells, CCL-2) and DF-1 (a continuous line of fibroblast cells, CRL-3586) cells were purchased from the ATCC. The cells were cultured in Dulbecco’s Modified Eagle Medium (DMEM; Gibco, Waltham, MA, USA) supplemented with 10% fetal bovine serum (FBS; Gibco, USA), 100 U/mL penicillin, and 100 µg/mL streptomycin (Gibco, USA) at 37 °C in a 5% CO_2_ humidified atmosphere. NDV strain Herts/33 was obtained from Dr. D. J. Alexander (Animal Health and Veterinary Laboratories Agency, Thirsk, UK) and propagated in specific-pathogen-free embryonated chicken eggs (Vital River, Beijing, China).

### 4.2. siRNA Sequence and Transfection

SiRNAs targeting various ESCRT components and si-NC were designed and synthesized by GenePharma, Shanghai, China. The sequences of siRNAs are shown in Table S1. siRNA transfection was performed at a final concentration of 50 nM using Lipofectamine RNAiMAX (Thermo Fisher Scientific, Waltham, MA, USA) according to the manufacturer’s protocol.

### 4.3. NDV Infection and Titration

The cells were exposed to the Herts/33 strain of NDV at indicated MOIs for 1 h in serum-free DMEM. Following the incubation period, unattached viral particles were eliminated by washing the cells three times with PBS. The cells were then maintained in complete growth medium at 37 °C in a 5% CO_2_ environment. Post-infection, the cell samples underwent subsequent analyses. For the TCID_50_ assay, cell supernatants were serially diluted and added to DF-1 cells seeded in 96-well plates. After 72 h, cytopathic effects were observed, and virus titers were calculated using the Reed-Muench method [57].

### 4.4. Plasmids Construction and Transfection

To generate the lentiCRISPR-HRS plasmids, three pairs of small guide RNA oligos (sgRNAs) targeting different exons of the HRS genomic locus in HeLa cells were designed using the CHOPCHOP web tool (version 3.0.0, https://chopchop.cbu.uib.no/ (accessed on 2 April 2023)). These sgRNAs were then cloned into the lentiCRISPR v2 vector (Addgene, Watertown, MA, USA) as per the protocol on Dr. Feng Zhang’s website (https://zlab.bio/ (accessed on 21 May 2023)). Briefly, the vector was digested with BsmBI (New England BioLabs, Ipswich, MA, USA) following the manufacturer’s instructions and purified using the Qiaquick Gel Extraction Kit (Qiagen, Shanghai, China). The sgRNAs were annealed and ligated into the linearized vector with T4 ligase (Thermo Fisher Scientific, Waltham, MA, USA). For constructing the plasmid expressing HRS protein (PCAGGS-HRS), the HRS gene coding region was amplified from HeLa cell cDNA using specific primers. The amplified gene was then inserted into the EcoRI/XhoI site of the PCAGGS vector (HonorGene, Changsha, China) using the ClonExpress Ultra One Step Cloning Kit V2 (Vazyme, Nanjing, China), following the manufacturer’s instructions. The sgRNA sequences are provided in Table S2, and the primers for HRS amplification are F: 5′-CATTTTGGCAAAGAATTCATGGGGCGAGGCAGCGGCAC−3′, R: 5′-GGAAAAAGATCTGCTAGCTCGAGGTCGAATGAAATGAGCTGGGCCTCGC-3′. All plasmids were confirmed by sequencing.

Plasmid expressing Flag-tagged M protein was constructed in our previous study [2].

For transfection, HeLa cells at 40% confluency were transfected with the plasmids using the TransIntro EL Transfection Reagent (TransGen, Beijing, China), adhering to the manufacturer’s protocol.

### 4.5. qPCR

For detection of ESCRT and NDV NP mRNA, the total RNA was extracted from HeLa cells using the TRIzol reagent (TransGen, China) and reverse-transcribed into cDNA using the PrimeScript RT reagent kit (Vazyme, China). qPCR was performed using TransScript Green One-Step qRT-PCR Super Mix (TransGen, China) according to the manufacturer’s instructions on LightCycler 480 (Basel, Switzerland).

For detection of NDV genomic RNA, the method was performed as previously described [58]. Briefly, total RNA extracted from NDV-infected cells was quantitatively analyzed. Equal amounts of RNA (2 µg) from each sample were reverse-transcribed using the following primers:

F: 5′-CATTTTGGCAAAGAATTCATGGGGCGAGGCAGCGGCAC-3′, R: 5′-GGAAAAAGATCTGCTAGCTCGAGGTCGAATGAAATGAGCTGGGCCTCG-3′. Subsequently, 1 µL of cDNA from each sample was quantified using specific primer pairs.

All primers used for qPCR were listed in Table S3. The expression levels of target genes were normalized to GAPDH as an internal control. The relative quantification was calculated using the 2^−ΔΔCt^ method.

### 4.6. Western Blot Analysis

Cells were lysed in RIPA buffer (Beyotime, Shanghai, China) supplemented with protease and phosphatase inhibitors (Roche, Basel, Switzerland). Protein concentrations were measured using the BCA Protein Assay Kit (Thermo Fisher Scientific, USA). Equal amounts of protein were separated by SDS-PAGE and transferred to PVDF membranes (Millipore, Burlington, MA, USA). Membranes were blocked with 5% non-fat milk in TBST and probed with indicated primary antibodies. HRP-conjugated secondary antibodies were used, and signals were detected using the ECL detection system (TransGen, China). Mouse monoclonal antibodies against HRS, CHMP4A, CHMP4B, CHMP4C, and Flag were purchased from Santa Cruz, USA. The mouse monoclonal antibody against GAPDH was purchased from TransGen, China. The anti-NP mouse monoclonal antibody was kindly provided from Prof. Ding Chan (Shanghai Veterinary Research Institute, Chinese Academy of Agricultural Sciences, Shanghai, China).

### 4.7. IFA

HeLa cells seeded on glass coverslips (WHB, Wuhan, China) in 24-well plates were fixed with 4% paraformaldehyde, permeabilized with 0.1% Triton X-100, and blocked with 5% BSA in PBS. Primary antibodies against NDV NP and secondary antibodies conjugated with Alexa Fluor dyes (Thermo Fisher Scientific, USA) were used. Nuclei were stained with DAPI (Beyotime, China). Fluorescence images were captured using a confocal laser scanning microscope (Leica Microsystems, Nussloch, Germany).

### 4.8. Generation of HRS Knockout HeLa Cells Using CRISPR-Cas9-Mediated Genome Editing

HRS knockout HeLa cell lines were generated using the CRISPR-Cas9 system. HeLa cells cultured in a 6-well plate were transfected with lentiCRISPR-HRS plasmids at approximately 80% confluence. The cells were selected with puromycin (2 µg/mL) for two weeks and subsequently serial-diluted to obtain a monoclonal cell line. The generated HeLa-HRS-KO cell lines were further confirmed by Western blot analysis.

### 4.9. Cell Viability Assay

Cell viability was assessed using the Cell Counting Kit-8 (CCK-8, Beyotime, China). HeLa cells were seeded in 96-well plates and transfected with indicated siRNAs. After 48 h, CCK-8 reagent was added to each well, and absorbance was measured at 450 nm using a microplate reader (Bio-Rad, Hercules, CA, USA).

### 4.10. LDH Release and Apoptosis Assay

HeLa-WT or HeLa-HRS-KO cells were seeded in 96-well plates for 12 h. Then, the cells were infected with the Herts/33 strain at an MOI of 1 for another 12 h. Subsequently, LDH release was assessed using the LDH Cytotoxicity Assay Kit with WST-8 (Beyotime, China) according to the manufacturer’s instructions. The percentage of LDH release was calculated according to the formula: LDH release (%) = (Experimental LDH release − Spontaneous LDH release)/(Total LDH release − Spontaneous LDH release) × 100%.

For detection of the apoptosis rate, NDV-infected cells were trypsinized by non-EDTA trypsin and collected by centrifugation at 500× *g*, 4 °C for 5 min. Then, the apoptosis rate was measured by the AnnexinV-FITC/PI Cell Apoptosis Detection Kit (TransGen, China) according to the manufacturer’s instructions.

### 4.11. Viral Attachment and Internalization Assays

For viral attachment assays, HeLa-HRS-KO and wild-type HeLa cells were incubated with NDV at an MOI of 10 at 4 °C for 1 h. Unbound viruses were removed by washing with cold PBS. For internalization assays, cells were incubated at 37 °C for 1 h after attachment. Attachment or intracellular viruses were quantified by IFA, Western blot, and qPCR analysis as described above.

### 4.12. VLP Preparation and Purification

HeLa-WT or HeLa-HRS-KO cells were transfected with plasmids expressing FLAG-M proteins. The cell lysate and supernatant samples were collected at 48 hpt. The VLP in the supernatant preparation and purification as described in a previous study [41]. The cell lysate and purified VLP samples were immunoblotted with anti-FLAG and anti-GAPDH antibodies as described above.

### 4.13. Statistical Analysis

All experiments were performed in triplicate. Data are presented as mean ± standard SD. Statistical analyses were conducted using GraphPad Prism 8 software (GraphPad Software, Boston, MA, USA). A *p* value of less than 0.05 was regarded as statistically significant. NS means no significant difference, * *p* < 0.05, ** *p* < 0.01, *** *p* < 0.001, **** *p* < 0.0001.

## Data Availability

The data presented in this study are available on request from the corresponding author.

## Supplementary Materials

The following supporting information can be downloaded at: https://www.mdpi.com/article/10.3390/ijms251810060/s1.
